# Integration in action: four international case studies

**Published:** 2012-07-24

**Authors:** Susanne Bethge

**Affiliations:** Institute Health Economics and Healthcare Management, University of Applied Sciences Neubrandenburg (Germany)

## Background and structure of the report

As the title says, this report comprises four case studies of different integrated care projects all over the world and displays “integration in action”. The report gives a broad view of how integrated care can work and aims to find a superior structure of advices on what is necessary to help integration to be successful. It defines “key ingredients for progress with integration” on the example of four actual integrated care programs.

The report follows the “classical” article structure and is divided into introduction, study design and methods, the case study organization, result section and a final discussion part. The present review is following this structure to give a short impression and will end with a final recommendation and conclusion.

## Background and research question

The report was published by the Nuffield Trust, which is examining the strength and weaknesses of new forms of integrated care. The aim of Nuffield Trust within this project is to inform policy makers as well as the practice in the UK about possible “ingredients” for better integrated health programs.

Based on the following four integrated care programs these “ingredients” were developed:

- **Community Care North Carolina, US**: a government-funded network that aims to improve access and quality levels for Medicaid beneficiaries;
- **Greater Rochester Independent Practice Association, US:** an independent practice association in upstate New York;
- **Regionale HuisartsenZorg Huevelland, The Netherlands:** an organization providing support to GPs to deliver integrated diabetes care;
- **North Lanarkshire Health and Care Partnership, Scotland:** an NHS and social care partnership.

## Research method and case study organizations

The different projects where evaluated by semi-structured interviews with 9–15 experts each. The interviews were transcribed and analyzed. Furthermore, a systematical analysis of the structure of the programs was conducted to find possible influencing factors.

The four organizations are very well described by their characteristics. A detailed overview about the four systems is given at page 16 in the report. The whole report includes several graphics, figures and tables that summarize the separate steps and give an encompassing overview about the different topics addressed in the report and outline the findings.

## Results of the report

Six processes can be extracted in the report that are necessary for integration:

1. **Clinical integrative processes:** Related to the delivery of consistent and standardized clinical care to patients along the whole continuum of care.
2. **Informational integrative processes:** Describe the development of clinical and managerial information systems to support aligned practice across different care settings, to enable communication between clinical teams, to enable outcome measurement and performance management as necessary.
3. **Organizational integrative processes:** Recommends the development of governance arrangements within and between institutions, and the design of organizational structure to aid integration.
4. **Financial integrative processes:** Refer to examples as joint budgetary arrangements and payment systems across organizations that help to “integrate”.
5. **Administrative integrative processes:** Recommends administrative support (for example, shared human resource management and seconded staff) to assist small practices and build links with the parent case study organization.
6. **Normative integrative processes:** Identifying, communicating and to operationalize the shared vision, goals and values across individuals and organizations could be described as key elements for integration.

Next to the six integrative processes three groups of important external factors influencing integration are identified and shortly described within the report. These groups are summarized by the keywords national policy, legal and regulatory requirements, and local payment systems.

Lastly, the discussion section of the report provides an extensive overview about actual studies and findings related to the findings of the present report and in integrated healthcare research.

## Conclusion and recommendation

The reader has to be aware, that the evaluation is done from the NHS perspective to sum up results for the improvement of the integrated care developments in the UK and their national system. Nevertheless, the descriptions of the four different systems provide a fruitful insight into different efforts of overcoming the (system) related problems. Overall the strengths and positive characteristics of new integrated care programs can be extrapolated.

I would recommend this report to everybody who is interested in the way integrated care is working in different systems and which enabler and challenges can come up. The report allows the reader to occupy different perspectives when examining integrated care programs.

